# Pelvic Vascular Malformations and Recurrent Rectal Prolapse in a Patient With Maffucci Syndrome: A Diagnostic and Therapeutic Challenge

**DOI:** 10.7759/cureus.39095

**Published:** 2023-05-16

**Authors:** Peter Sciberras, Rebecca Calleja, Rebecca Bugeja, Gloria Montebello, John Camilleri-Brennan

**Affiliations:** 1 Surgery, Hull University Teaching Hospitals NHS Trust, Hull, GBR; 2 Surgery, NHS Greater Glasgow and Clyde, Glasgow, GBR; 3 Surgery, Northern Care Alliance NHS Foundation Trust, Salford, GBR; 4 Surgery, Mater Dei Hospital, Msida, MLT; 5 Surgery, NHS Forth Valley, Stirling, GBR; 6 School of Medicine, Dentistry and Nursing, University of Glasgow, Glasgow, GBR

**Keywords:** recurrent rectal prolapse, pelvic floor dysfunction, maffucci syndrome, laparoscopic surgery, haemangiomatosis syndromes, coloproctology

## Abstract

Maffucci syndrome is an extremely rare congenital condition characterized by the development of multiple enchondromas and haemangiomas, primarily on the extremities, and an association with various tumors. Colonic and pelvic floor function has never been explored in patients with Maffucci syndrome. We report a case illustrating the challenges in managing colonic and pelvic floor dysfunction in a female patient secondary to vascular malformations as part of Maffucci syndrome.

## Introduction

First described in 1881 by Italian pathologist Angelo Maria Maffucci, Maffucci syndrome is an extremely rare congenital condition of which only around 200 cases have been recorded in the literature [1,2]. It is an idiopathic congenital mesodermal dysplasia characterized by the development of multiple enchondromas and haemangiomas, primarily on the extremities [3], and is diagnosed on clinical, radiologic, and pathologic grounds [4]. Maffucci syndrome is part of a family of haemangiomatosis syndromes, members of which include Klippel-Trenaunay syndrome, Kasabach-Merritt syndrome, and Proteus syndrome [2]. Most affected individuals are normal at birth, with the typical skeletal and vascular lesions occurring before puberty. The average age at onset is four years [5,6]. Intelligence is unaffected, and individuals with Maffucci syndrome achieve normal developmental milestones [7]. There is also an association with other types of vascular growths [1] and various benign and malignant tumors, including lymphangiomas, gliomas, ovarian fibrosarcoma, and acute myeloid leukemia [8]. The main focus of the published literature on patients with Maffucci syndrome and other related overgrowth syndromes has been on diagnosing and treating the characteristic malformations and associated malignancies. Other aspects of these patients’ quality of life, such as colonic and pelvic floor function, have received scant attention. This paper reports the case of a patient with Maffucci syndrome who suffered from recurrent rectal prolapse associated with haemangiomas. This article was previously presented as a poster at the 17th European Society of Coloproctology (ESCP) scientific conference in Dublin, Ireland, on September 21, 2022.

## Case presentation

**Figure 1:**
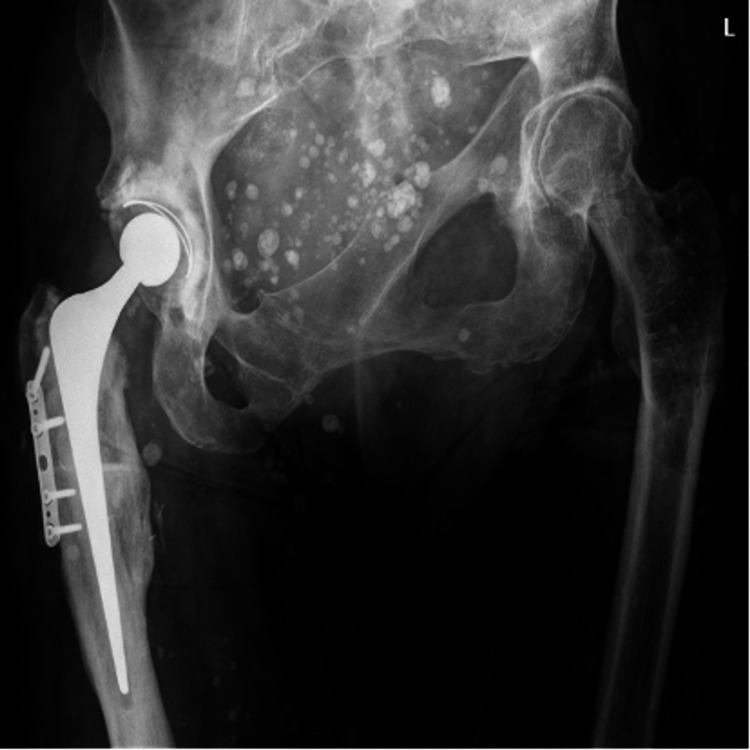
Frontal radiograph showing the extensively deformed pelvis and right total hip arthroplasty.

We introduce the case of a 64-year-old white nulliparous female who initially presented to our unit in October 2010. She was diagnosed with Maffucci syndrome at the age of two when she was noted to have multiple angiomata and bony periarticular growths. She required several orthopedic interventions in childhood, including a left through-knee amputation at the age of seven, resulting in her pelvis becoming misaligned and deformed by the time she reached adulthood (Figure 1). 

**Figure 2:**
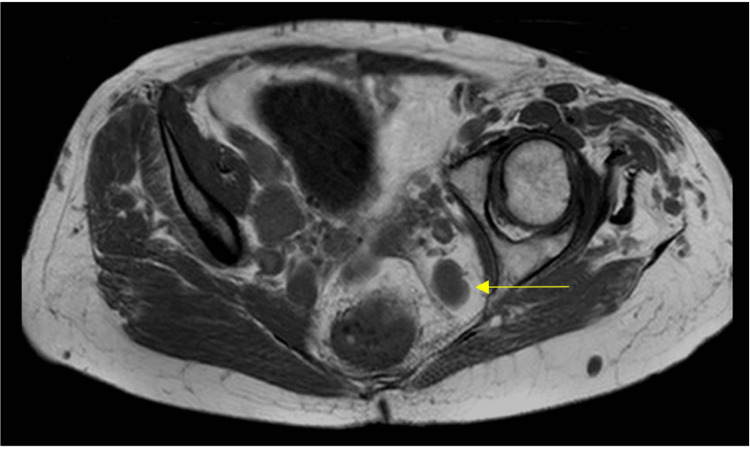
Transverse section of pelvic MRI prior to the first trans-anal repair procedure, showing one of the larger vascular malformations (arrow).

In 1999 she had a vaginal hysterectomy for menorrhagia. This was followed, 10 years later, by a tension-free vaginal transobturator tape (TVT-O) and anterior colporrhaphy for stress urinary incontinence and a cystocele. These procedures were only moderately successful in controlling her urinary symptoms. The patient’s presenting symptoms were a reducible anal lump and fecal incontinence, and she was clinically diagnosed with a circumferential lower third rectal mucosal prolapse. MRI also revealed the presence of several vascular lesions within the pelvis, the largest being 3.6 cm in diameter (Figure 2). The rectal mucosal prolapse was treated with injection sclerotherapy, albeit unsuccessfully. 

**Figure 3:**
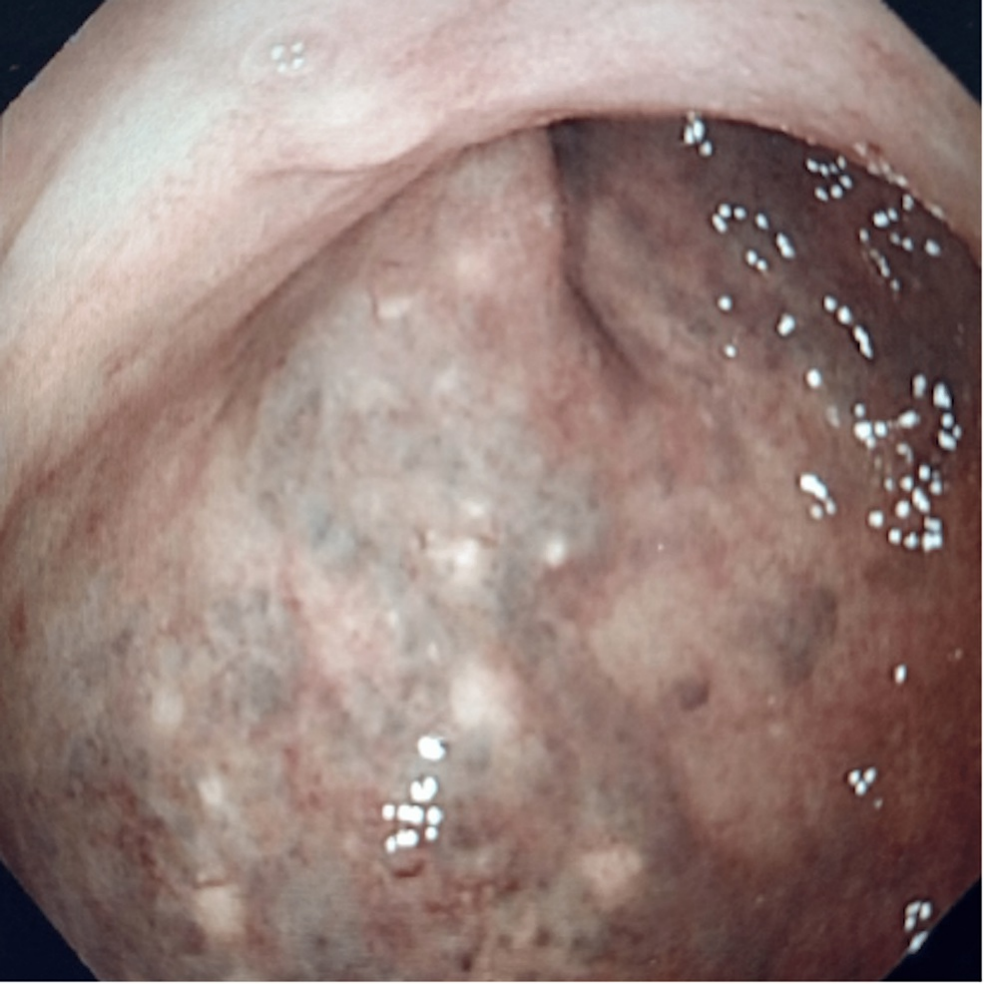
Dilated tortuous submucosal rectal veins typical of Maffucci syndrome on sigmoidoscopy.

Following a period of conservative management, during which she also underwent various orthopedic operations, including a right hip arthroplasty for secondary osteoarthritis, she was diagnosed with an anterior rectocele in 2012. Her primary symptoms at the time were slow transit constipation, obstructive defecation, and a degree of passive fecal incontinence. Interestingly, she was noted to have submucosal rectal varicosities on sigmoidoscopy (Figure 3). Her symptoms were managed with laxatives and rectal irrigation. Despite this, her ability to defecate unaided gradually worsened, and, following further assessment, she had a trans-anal rectocele repair a year later. The excised prolapse was rich in submucosal varicosities and lipomata. Histology showed several hard, smooth cream nodules beneath the mucosa ranging from 5 mm to 8 mm in diameter. Embedded in the submucosa were numerous medium-to-large caliber vessels, some of which contained amorphous eosinophilic material, which in places showed calcification arranged in a lamellar fashion. These histological features are in keeping with a haemangioma with thrombosis formation.

**Figure 4:**
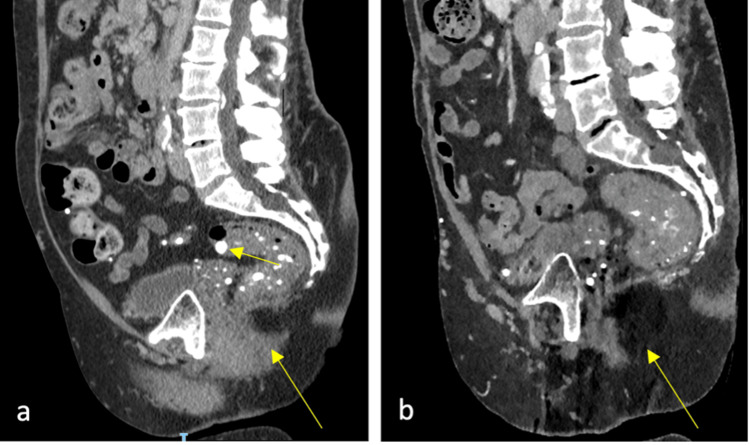
Sagittal sections of pelvic CT before (a) and after (b) Hartmann's operation, showing several vascular lesions within the pelvis (small arrow) together with the rectal stump in situ and resolution of the rectal prolapse postoperatively (large arrow).

The success of the trans-anal rectocele repair was short-lived, however, and her obstructive defecatory symptoms recurred within a few months. At this stage, the mainstay of her management was under the guidance of the physiotherapy team, using pelvic floor exercises and rectal irrigation on a daily basis, but her symptoms progressed nonetheless. Two years later, after suffering from increasing upper abdominal discomfort, she was diagnosed with a necrotic mixed solid and cystic mass in the uncinate process of the pancreas, with multiple venous collaterals and varicosities around the duodenum and the head of the pancreas. It was decided to manage this tumor conservatively, given that it was confirmed to be benign on histological examination. On further colorectal follow-up in 2015, she was diagnosed with a recurrent rectocele and a rectal mucosal prolapse. Following a failed trial of conservative management, she went on to have another trans-anal repair in August 2017. This operation, combined with postoperative physiotherapy, relieved her from her symptoms for a few months. After a hiatus of less than two years, the patient presented with fecal urgency and urge fecal incontinence attributable to a low rectal prolapse. Following extensive multidisciplinary discussion, it was decided to carry out a laparoscopic rectopexy, given that trans-anal operations had failed and her recurrences were progressively worsening and limiting her quality of life. Given the patient's extensive dilated pelvic veins, we knew that laparoscopic rectopexy would carry a high risk of intraoperative bleeding. Therefore, following comprehensive counseling, the patient consented to a laparoscopic ventral rectopexy with the possibility of sigmoid colon resection and stoma formation. Surgery, postponed due to the COVID-19 pandemic, was eventually carried out in August 2021. At laparoscopy, she was noted to have a redundant loop of sigmoid colon 50 cm long, packed with small hard pellets of stool, which were also present in the transverse colon. Multiple pelvic adhesions, liver scarring, and dilated mesenteric and pelvic veins were noted, making surgery challenging due to the high risk of inadvertent organ injury and hemorrhage. Given these conditions and the distended nature of the colon and impacted stool, combined with the high risk of recurrence, we decided not to proceed with a laparoscopic rectopexy and instead carry out a sigmoid colectomy with an end colostomy fashioned in the left iliac fossa (Figure 4). 

The patient had an uneventful recovery and was discharged after five days. She was, however, readmitted on day seven postoperatively with peritonitis secondary to a distal colonic stercoral perforation of the stoma at the abdominal wall level. Further colon was resected, and a transverse end colostomy was fashioned. At the follow-up consultation, she was noted to have stomal prolapse, for which refashioning was successfully carried out in April 2022. At her most recent clinic visit, she reported that her stoma was working well and denied any issues attributable to rectal prolapse or pelvic floor dysfunction.

## Discussion

The surgical options for rectocele and rectal prolapse are diverse. The choice ultimately depends on the clinical status of the patient and the surgeon's preference and experience [9]. Patients usually undergo a trial of conservative management before being considered for more invasive treatment options. Perineal approaches such as Delorme's and Altemeier's procedures are anecdotally associated with fewer postoperative complications and a quicker recovery but higher recurrence rates. They are preferred in elderly frail individuals, with abdominal operations reserved for patients with a more favorable risk profile [9-12]. Treatment aims to control the prolapse, improve defecatory function [9,13], and reduce the risk of complications, including rectal incarceration or even strangulation [14]. Gastrointestinal (GI) vascular lesions are rare in patients with Maffucci syndrome. Few such reports exist in the literature, with the dominant features of the disease being cutaneous and musculoskeletal growth. The three primary vascular malformations seen with Maffucci syndrome are cavernous haemangiomas, phlebectasia, and lymphangiectasias-lymphangiomas [5]. These have been found throughout the entire GI tract, including the oral cavity [15], pharynx [16], small bowel [17], ascending colon [5], and rectosigmoid area [18], with the most notable symptoms being bleeding and iron-deficiency anemia. Shepherd V et al. previously reported a case of a nine-year-old female with Maffucci syndrome requiring three emergency laparotomies for bleeding or intussusception [7]. Interestingly, our patient had evidence of such GI involvement in the form of widespread venous malformations throughout the pelvis and in the rectum. This is likely to have resulted in laxity and weakening in the rectal supporting tissues, which, together with the raised pelvic pressure from significant venous congestion, predisposed to recurrent rectal prolapse resistant to treatment (Figure 3). After thorough searches of the MEDLINE and Embase databases, this is, to our knowledge, the first report in the literature describing rectal prolapse as the result of a vascular malformation in a patient with a diagnosis of Maffucci syndrome or any other haemangiomatosis syndrome. 

Trans-anal approaches were adopted initially in this case, as there was a reluctance to carry out major abdominal surgery in the form of laparoscopic rectopexy as a primary procedure due to the perceived high risk of bleeding. The success of such approaches was short-lived, however. Around 30% of surgeries for primary rectal prolapse are unsuccessful, particularly in females [10]. The high recurrence rates are likely due to technical failure and inadequately controlled patient factors [11-13]. We believe that the presence of soft tissue growths and pelvic varicosities, previous hysterectomy, a deformed pelvis, weak pelvic floor musculature, chronic constipation, and straining all contributed to the patient's pelvic organ prolapse. These same factors may have also been responsible for the lack of long-term success of her operations and conservative approaches involving physiotherapy. Given the adverse conditions within the colon and pelvis, our decision during the operation was that a laparoscopic sigmoid colectomy with an end colostomy was likely to be a safer and more effective alternative to ventral mesh rectopexy. Although this operation was beset with early complications that necessitated revisional surgery, the long-term outlook for this patient is promising. In fact, a year down the line, she is managing her stoma well and is experiencing a marked improvement in the colorectal aspect of her quality of life.

## Conclusions

This case report highlights the difficulties encountered in the decision-making and management of slow transit constipation and pelvic floor dysfunction in a female patient with Maffucci syndrome. The presence of the various associated malformations had initially led us to pursue a more cautious and measured approach. Nevertheless, given the numerous recurrences of pelvic organ prolapse in this patient, we believe that patients with similar overgrowth syndromes and aberrant anatomies would be less likely to respond to conservative management and should therefore be considered for invasive treatment strategies at an earlier stage to reduce the risk of recurrence. A multidisciplinary approach is key in managing recurrent rectal prolapse, with decisions made on an individual basis after thorough patient counseling.
